# Parental participation during children’s vital sign monitoring in connection with surgery for congenital heart disease

**DOI:** 10.3389/fped.2026.1809877

**Published:** 2026-06-09

**Authors:** Carina Sjöberg, Annika Zetterberg, Helene Åvik Persson, Karina Terp, Pether Jildenstål

**Affiliations:** 1 Department of Health Sciences, Faculty of Medicine, Lund University, Lund, Sweden; 2 Department of Anaesthesiology, Surgery and Intensive Care Medicine, University Hospital, Gothenburg, Sweden; 3 Faculty of Nursing and Health Sciences, Nord University, Bodö, Norway; 4 Department of Anesthesiology and Intensive Care, Skane University Hospital, Lund, Sweden; 5 Department of Anesthesiology and Intensive Care, Örebro University Hospital and School of Medical Sciences, Örebro, Sweden; 6 Institute of Health and Care Sciences, Sahlgrenska Academy, University of Gothenburg, Gothenburg, Sweden

**Keywords:** children, congenital heart disease, monitoring, monitoring parent participation, vital signs

## Abstract

**Background and purpose:**

The experience of having a child monitored for vital signs in relation to surgery for congenital heart disease can be highly stressful for parents. The opportunity to be involved in their child's care is central to parents’ well-being and to their overall experience of the care provided. The purpose of this study was to explore how parents of children with congenital heart disease experience and perceive their participation during pre- and postoperative monitoring of vital signs.

**Design and methods:**

A qualitative exploratory study with purposive sampling was conducted. Ten parents of children aged 3–36 months who underwent congenital heart surgery were included. All had experience of high-technology care environments during pre- and postoperative monitoring. Data was collected through semi-structured interviews between March 2024 and January 2025 and analyzed using qualitative content analysis.

**Results:**

The parents’ experiences and perceptions of their participation are described in one major theme striving for connection and control through participation and support in a monitored care environment. This theme is further reflected in three categories: “information as a foundation for parental participation,” balancing the need for proximity and separation, and “support for family presence and participation,” each with their respective subcategories.

**Conclusions and implications:**

Parents’ experiences of participation are influenced by the extent to which healthcare professionals involve them through tailored information and individualized adaptations. Parents require support to remain close to and assume responsibility for their child, while also feeling confident in entrusting aspects of care to healthcare professionals.

## 1 Introduction

Congenital heart disease (CHD) is one of the most common congenital conditions, affecting approximately 1% of all live births worldwide and representing a major contributor to early-life morbidity (1–3). This incidence is consistent with Swedish data, where approximately 1,000 children are born each year with a cardiac malformation. Many of these children require surgical intervention early in life, contributing to the population of young children undergoing surgery (4). Advances in surgical techniques and perioperative care have led to improved outcomes over time in high-income countries, with increased survival, 90% survive up to five years of age, and approximately 85-90% of those children reach adulthood. However, prognosis varies significantly, particularly in those with complex cardiac defects, where long-term morbidity and mortality remain increased compared with the general population (5–8). For many children with CHD, surgery aims to correct rather than cure the defect, and those with complex cardiac anomalies often require long-term specialized care and continuous follow-up. Regular assessments and additional medical or surgical interventions are frequently necessary throughout childhood and later in life (4).

In Sweden, children with complex medical conditions, including those undergoing cardiac surgery, receive perioperative care in pediatric intensive care units (PICUs) located at four national tertiaries care centers, two of which provide specialized surgical care for children with CHD. Pediatric intensive care in Sweden is characterized by high staff-to-patient ratios, with critically ill children commonly receive one-to-one nursing care, continuous advanced monitoring, and access to specialized life-support treatments. Care is delivered by multidisciplinary teams with pediatric expertise, reflecting the highly centralized and resource-intensive structure of Swedish PICUs (9). The PICU environment can be overwhelming for parents, characterized by complex technical equipment, bright lights, and constant sounds from monitors and alarms. Parents often describe this environment as both frightening and confusing (10, 11). It can be deeply distressing for parents to see their child connected to advanced monitoring devices, to observe sudden alarms or changes, and to process the medical interventions or, in some cases, the absence of interventions (11–13). For example, many parents report being afraid to touch or comfort their child for fear of disturbing monitoring equipment or intravenous lines (14, 15).

The sounds and visual appearance of alarm systems used in medical equipment are likely to influence how parents perceive and experience the care environment in pediatric intensive care units (16, 17). Parents' sense of safety and participation is also closely linked to the establishment of trusting relationships with healthcare professionals (14, 18, 19). In this context, the presence of advanced monitoring technology has been shown to shape parents’ emotional experiences and their perceived parental role during their child's intensive care (10, 12). Research in pediatric intensive care settings indicates that routine monitoring, such as electrocardiograms (ECGs) and respiratory measurements displayed on bedside monitors, significantly influences parents' experiences and their relationships with both their child and the healthcare team (20). Healthcare professionals can promote parents' participation and help them feel included in the situation with information and explanations about the equipment, monitors, tubes, and drains, which have been shown to reduce fear and enhance their understanding of the care provided (19). Although pediatric intensive care units aim to promote parental participation through a family-centered care approach, still challenges remain in parents' opportunities to participate in their child's care (12, 21).

The principles of family-centered care constitute a model that emphasizes respect, dignity, participation, information sharing, and collaborative partnerships between healthcare professionals and families. Within this framework, parental participation refers to parents being actively engaged in both decision-making and the practical aspects of their child's daily care (22). Even though a family-centered care approach has gained increasing emphasis in pediatric settings (23). In a qualitative synthesis, McMahon and Chang (24) highlight the need for a deeper understanding of parents’ experiences to optimize support and reduce parental stress when caring for infants with CHD. While parenting a child with CHD undergoing surgery and intensive care is known to involve multiple stressors, the specific contribution of pre- and postoperative monitoring to parental stress remains unclear and warrants further investigation (25). A sense of parental control is central to health (22). When parenting a child with CHD, parents need to feel safe with the situation and the clinical monitoring of their child's vital signs, while retaining a sense of participation and control. One of the core concepts in family-centered care is participation, meaning that families are encouraged and supported to participate in care and decision-making at the level they choose (22).

In this context, this means that parents feel safe and comfortable in the high-tech care environment, supporting their ability to participate and function as parents to their child. Therefore, this qualitative exploratory study aimed to explore how parents of children with congenital heart disease experience and perceive their participation during pre- and postoperative monitoring of vital signs.

## 2 Methods

### 2.1 Design

This study employed a qualitative, exploratory design with an inductive approach to explore parents' experiences and perceptions (26, 27). Data was gathered through semi-structured individual interviews. A qualitative content analysis, as described by Graneheim and Lundman (28) was used to identify patterns, variations, differences, and similarities in the parents’ perceptions (28).

### 2.2 Setting and participants

This study was conducted at one of the Swedish national healthcare centers specializing in the treatment and surgical care of pediatric patients with CHD. Inclusion criteria comprised parents (one per child) of full-term infants aged 3–36 months who had experience of their child's care, including pre- and postoperative vital sign monitoring in connection with surgery for CHD. Exclusion criteria included insufficient proficiency in spoken Swedish to participate in an interview, being a minor, and hearing impairment (i.e., deafness).

Participants were recruited using purposive sampling (one parent per child). The sample comprised 10 parents of children undergoing cardiac surgery with pre- and postoperative vital sign monitoring.

### 2.3 Data collection

Parents were initially informed about the study verbally by a contact nurse during the preoperative scheduling phone call and were asked about their interest in participation. For those expressing interest, contact details were shared with AZ, who subsequently provided additional information by telephone. Written study information was then sent by post, allowing parents time to consider participation.

Parents who agreed to participate were scheduled for postoperative digital interviews. Verbal consent was obtained before the interview, and written informed consent was returned by post before any data were included in the analysis. In connection with the interviews, data on the participating parents' sociodemographic characteristics were collected, along with medical data on the child, including age, sex, and reason for admission (Table 1).

**Table 1:** Parentś and children's characteristics.

Demographic Characteristic Parents (*n* = 10)	
Mothers	9
Fathers	1
Age years (range, mean)	27–42 (36)
Prior PICU monitoring experience^a^	4
**Education**	
High school	2
Higher vocational education	3
College/University	5
Demographic Characteristics Children (*n* = 10)	
Age month ^b^(range, mean)	3-36 (15)
Male	6
Female	4
**Admission cause**	
Tetralogy of Fallot	4
Ventricular septum defect (VSD)	3
Mitral regurgitation	1
Coarctation of the aorta	1
Sinus venosus atrial septal defect	1

a Prior PICU monitoring experience: Previous experience of the child being monitored for vital signs.

b Full-term infants from 3 to 36 months.

Individual interviews were conducted using a semi-structured interview guide on parents' participation in their children's care, which was applied consistently to all participants. The interview guide was originally developed for a study on parents' participation in pediatric perioperative care (29) (see Supplementary Material S1). A total of 10 interviews were conducted between [March 2024] and [January 2025]. The interviews were conducted within 14 days of the parents' and child's discharge from the hospital. All interviews took place via Zoom with audio recording only. The audio was recorded using an external device and transcribed verbatim. The interviews were conducted by AZ and began with questions about sociodemographic and medical background. This was followed by the opening question: “Can you tell me about your experience with your child being monitored using medical technology before and after surgery?”

The interviews proceeded with open-ended and follow-up questions to allow participants to express themselves freely and in their own words. Toward the end of each interview, a summary was provided, allowing participants to confirm or clarify the interviewer's interpretations. The interviews lasted between [10] and [26] minutes. Data collection continued until data saturation was reached, when no new information emerged (30). To assess the clarity and relevance of the interview guide, a pilot interview was conducted. Since no revisions were necessary, the data from this interview were included in the final analysis.

### 2.4 Ethical considerations

Ethical approval was granted by the Swedish Ethical Review Authority (ref. 2022-00896-01). The study complies with the Helsinki Declaration, and all informants have been informed and have given consent.

### 2.5 Data analysis

The interviews were analyzed according to Graneheim and Lundmans qualitative content analysis (28), using an inductive approach. The analysis began with a thorough reading of the interviews, by all authors to gain an overall impression of their content. Thereafter, the text was divided into meaning units (sentences and paragraphs) related to the aim of the study by two of the authors (C.S, A.Z). Meaning units were then extracted and condensed from the transcripts, i.e., the text was shortened while the essential parts were preserved. Next, the condensed meaning units were labelled with a code by four of the authors (C.S, A.Z, H.Å-P, K.T) independently. Four of the authors (C.S, A.Z, H.Å-P, K.T) read the codes repeatedly and compared them to determine their corresponding content and then grouped accordingly. Codes with corresponding content were compiled into subcategories and were adjusted as needed. Similar subcategories were then grouped into the main categories. In the final step, the latent content was summarized into a theme. The analysis process is illustrated through examples presented in Table 2.

**Table 2:** Example of the analysis process.

Meaning unit	Condensed meaning unit	Code	Subcategory	Main category	Theme
We received information all the time, I think continuously, so we received that information over and over again so that… well, but so that we would simply remember it and be involved.	Received information all the time, I think continuously.	Information	Being kept informed about procedures and actions	Information as a foundation for parental participation	Striving for connection and control through participation and support in a monitored care environment

The analysis process was iterative in nature, involving a continuous co-creation between the authors and the text. All authors independently reviewed the data and discussed discrepancies until consensus was reached, resulting in six subcategories, three main categories, and the overarching theme. Quotations from the interviews were chosen and added to ensure transparency, preserve and support meaning, and to exemplify each sub-category. All interviews were conducted by the second author (A.Z), a registered nurse anesthetist and pediatric nurse specialist, who had no prior relationship with the participants. Awareness of the pre-understanding was employed throughout all the interviews to prevent influence on the follow-up questions. Throughout the analysis process, the authors engaged in continuous reflexive discussions and reflections to prevent their pre-understanding might influence the data analysis. These discussions were used to reduce the influence of preconceptions and to strengthen the credibility of the findings.

## 3 Result

The analysis identified one major theme, along with three main categories and six subcategories (Table 3), which together illustrate how parents experience participation in the context of their child´s pre- and postoperative monitoring vital signs.

**Table 3:** Overview of the theme, main categories, and subcategories from the analysis.

Theme	Striving for connection and control through participation and support in a monitored care environment		
Main categories	Information as a foundation for parental participation	Balancing the need for proximity and separation	Support for family presence and participation
Subcategories	Preparatory information to understand procedures and monitoring Being kept informed about procedures and actions	Monitoring provides a sense of control Distance creates emotional distress	Support for siblings and relatives Facilitating access and inclusion in care

### 3.1 Striving for connection and control through participation and support in a monitored care environment

The overarching theme identified that parents struggled to balance participation with circumstances, feeling that they were trying to maintain a sense of closeness and control in a monitored, technology-driven context. Reassurance and support from healthcare professionals play a significant role in parents' ability to participate in their child's care.

### 3.2 Information as a foundation for parental participation

The parents expressed a need for clear, continuous, and accessible information during the perioperative period, to support their understanding of clinical procedures and the monitoring of their child. This involved both preparatory information and ongoing information within the monitored care period, especially during clinical challenges, enabling parents to remain informed about their child´s condition and ongoing monitoring. Parents emphasized that receiving adequate information not only reduced their concerns but also allowed them to participate actively in their child's care.

#### 3.2.1 Preparatory information to understand procedures and monitoring

Parents highlighted the importance of receiving information before the surgery, including written materials, visual aids, and explanations about what to expect. Such preparation was crucial, as it helped parents anticipate the postoperative environment, understand the medical equipment, and mentally prepare for their child's condition. “And that was really good, because they prepared everything very well and showed us pictures of what it would look like after the operation.” (3)

Being well prepared increased the understanding of the procedure and made it less shocking to see them in the high-technology environment of intensive monitoring, connected to multiple wires and tubes. “They had visual materials that they went through with us, along with other children, and it was necessary; otherwise, it would have been somewhat shocking.” (8) Some parents reported that although they knew what to expect, seeing it was still somewhat frightening, while others focused less on the wires and tubes and perceived their child mostly as peacefully sleeping. “I was still quite prepared for her to have a lot of wires and tubes and to be sedated, and it looked very peaceful - it looked like she was just sleeping.” (1)

For some parents, the discrepancies between preparatory information and the actual course of care led to anxiety. For example, when the parents were told that the child would stay for approximately two weeks, and when this changed, it was difficult for them to adjust their understanding of the care process and the timing of discharge. Others interpreted information about the intensive monitoring in the PICU unit, meaning they would not be allowed to stay with their child during that period, which they experienced as frightening. “It makes you absolutely terrified, partly because you have to send your child off for heart surgery and leave her there, and then you’re not allowed to be with her. And then there's the fact that she's not allowed to have her mum there, you know, I mean, that was really tough.” (8).

#### 3.2.2 Being kept informed about procedures and actions

Ongoing access to information, including updates from healthcare professionals, and the ability to ask questions and receiving regular explanations of the child's condition were crucial. Parents expressed that ongoing communication, and clear, consistent explanations helped them feel informed and included in decisions, reassured, and able to cope with the unfamiliar hospital-monitored environment and moderate feelings of fear or uncertainty. “I think it was mainly because we were given plenty of information and knew exactly what was going on at all times. So, I believe that if we hadn't been given that, it would probably have been scarier to see our child connected to all sorts of things.” (2) Parents perceived receiving explanations and being informed about why monitoring alarms were triggered or why certain post-operative events were common, was particularly reassuring because it helped parents interpret alarm signals as non-critical. Although parents did not always know the precise meaning of each alarm, they felt encouraged to ask questions at any time. If healthcare professionals on the ward were unable to answer immediately, they consulted a colleague or obtained information to respond to the parents. “…as soon as we asked a question, even if they didn't know, they asked someone else who could answer“ (3).

Parents emphasized how their child experienced an abnormal heart rhythm after surgery, which led to increased monitoring and additional examinations. This initially caused stress, but information and explanations about the purpose of monitoring calmed the parents. However, parents expressed that they sometimes wished they had received more information, especially when changes appeared in their child's monitoring. “Above all, you need information, you want to know what's happening and how your child is doing, because it's not always clear whether you really understand. It often looks quite bad.” (5).

Delays in information or inconsistent communication sometimes left parents to guess or interpret data themselves, which increased stress. Instead of having to interpret for themselves what these changes meant, parents would have preferred to be informed that such variations are common, for example, occurring in approximately one-fourth of children after this type of surgery. This kind of information would have been more reassuring than leaving them to interpret the situation by themselves. “I’d really like to know what happened and why it beeped, or why he did this and that, instead of just sitting here guessing.” (9)

### 3.3 Balancing the need for proximity and separation

Parents experienced the highly monitored hospital environment as both reassuring and challenging. Continuous monitoring gave them a sense of control, yet alarms, tubes, and equipment could provoke emotional distress, such as anxiety. Some parents felt that monitoring and the complexity of the equipment created distance from their child, making physical closeness difficult. Consequently, experiences of how parents negotiated closeness and separation while adapting to the high-technological care environment was highlighted.

#### 3.3.1 Monitoring provides a sense of control

Parents highlighted that continuous monitoring of their child's vital signs created a strong sense of security. Being able to observe oxygen saturation, heart rate, and other physiological parameters reassured them that their child's condition was being closely supervised, even when direct physical intervention was impossible. The presence of competent healthcare professionals who explained the purpose of each device and the meaning of alarms further strengthened parents' understanding and involvement. Over time, parents became familiar with the equipment and learned to interpret the monitors, which reduced uncertainty and enhanced their confidence in the care being provided. “…you quite quickly learn what all the beeps mean, and what all the devices do, which also makes you a bit less worried afterwards, …” (5). Parents acknowledged that monitoring was necessary, even when the child was connected to multiple lines and devices, and it reassured them that any deviation from normal parameters would be addressed. “I think it feels quite safe because then you know that they have an eye on the situation, that is, what is happening inside the body, what you can't see.” (2).

The combination of feedback and professional guidance, as well as repeated explanations, allowed parents to focus on emotional support for their child, generating reassurance and adaptation to the high-technological care environment, rather than being overwhelmed. “It's obviously really tough to see your child like that, but the monitoring part itself felt very safe and good. Very knowledgeable staff and all those parts” (5). Parents consistently expressed a strong need for unrestricted access to their child to prevent feelings of loss of control. When access was limited, for instance, due to space constraints or monitoring, parents experienced frustration and partial control. “Yes, I was able to be there all the time and had the opportunity to hold him. And we absolutely got that; when he was upset and needed to be held, they helped us lift him into our arms. And of course, it was difficult to keep all the equipment in place.” (7).

#### 3.3.2 Distance creates emotional distress

Initially, leaving the child was experienced as emotionally difficult, and the technical nature of monitoring sometimes created a sense of emotional distress for the parents and awoke feelings such as anxiety. Parents felt restricted in holding or comforting their child because of the equipment attachments, which also could provoke anxiety, despite the reassurance offered by monitoring. Unexpected alerts or irregular values served as constant reminders of the child's vulnerability, and parents sometimes felt powerless because they could not intervene. “Then it’s clear that you react, that it’s clearly a very, very difficult situation and above all it’s difficult to see him feeling bad, and then it’s difficult with all the tubes and wires and everything and stuff that goes with these pieces.” (5).

In some situations, assistance from healthcare professionals was required to lift or reposition the child, underscoring their limited ability to provide direct care. Although parents understood the necessity of monitoring vital signs, the physical barriers and technical interventions highlighted the separation between them and their child, creating feelings of frustration and helplessness.“…I didn’t really know how to lift [the child],… I’m not used to it at all and was just afraid that I might pull out a tube or something…” (1).

Parents negotiating between respecting the need for monitoring and maintaining closeness with their child. Over time, as they became more accustomed to the devices and received guidance from healthcare professionals, some parents reported that they could engage more comfortably. They found ways to hold or interact with their child while still maintaining safety with healthcare professionals assisting them. “Just that I got to be close. So, nothing else was important” (8). Although closeness was prioritized, parents also recognized the necessity of taking breaks to rest, eat, or regain strength, maintaining their own well-being, trusting that the child was safe in professional care. “Then it was quite nice to be able to go out for a while and, as a parent, be able to breathe for a few minutes, just to get away and eat and then be able to come back.” (2).

### 3.4 Support for family presence and participation

Parents emphasize the importance of receiving professional support to feel safe, informed, and included in their child's care in a monitored care environment. This support was not only important for their own sense of security but also influenced by how comfortable siblings felt, how involved they could be in care, and their ability to participate in care procedures, even within a monitored care environment.

#### 3.4.1 Support for siblings and relatives

Parents perceived that support for siblings and other relatives was important not only for the child's well-being but also for the family's overall ability to cope with the intensive care experience. Some parents were unaware of the available sibling support during the first week, which led to feelings of dissatisfaction and missed opportunities for guidance. “Maybe someone could have explained better to him what was happening, so that he could have at least asked questions to the nurse. Because I think he was probably mostly afraid of his little sister.” (3). They highlighted that siblings often had questions or worries about the hospitalized child, and structured support could help them understand the situation and feel involved. “The only feedback I had … they had sibling supporters that I didn’t know about during the first week” (3).

At the same time, parents expressed that support for themselves as relatives was limited, especially regarding emotional needs. They described proactive outreach from healthcare professionals, such as a psychologist checking in, was highly valued, while having to initiate such contact by themselves were challenging during stressful periods. “Yes, but that would have been, to get a little more…help for us as parents with just like mental, or those pieces, like how to cope in the best way and be able to support each other in the best way” (5). Parents emphasized that this type of support helped them manage their own stress, stay emotionally available for their child, and maintain a sense of control in an unfamiliar and monitored care environment. “A psychologist came and tapped me on the shoulder and said: ‘Hi, shall we talk a bit?” (5).

#### 3.4.2 Facilitating access and inclusion in care

Being actively involved in procedures allowed parents to feel competent, reassured, and deeply connected to their child. Participation ranged from small practical tasks, such as helping with feeding, adjusting equipment, or holding the child during procedures, to being present during rounds and discussions about care decisions.

“And then you could help and attach some sticky notes to the ECG that came loose, or there were some wires that you had to untangle, you got to be involved” (2). The parents valued it when healthcare professionals allowed enough time for explanations, decision-making, and for the child to adapt to procedures. The fact that healthcare professionals had reduced stress and created trust. Parents emphasized that these opportunities strengthened their role as essential caregivers and strengthened parent-child bonding, providing comfort in a monitored care environment that could otherwise feel highly technical and intimidating.“…the greatest need was probably to be there all the time, not being prevented from being there at any time but being able to be there exactly when we wanted and needed” (2).

Inclusion was experienced not only through being invited to perform tasks but also by being encouraged to stay near the child, to ask questions, and to be part of decision-making. “Then I think the healthcare professionals are good at saying that we are the parents, we know best, as well” (6). When healthcare professionals recognized the parents' knowledge about their child's needs and preferences, it created a sense of respect and competence. This acknowledgment was fundamental for reducing feelings of helplessness and empowering parents to provide emotional and practical support to their child. The consistent support and reassurance from healthcare professionals helped parents feel that their presence was meaningful. Parents felt included when healthcare professionals asked about familiar routines, comfort items, or ways to calm the child, confirming their parental knowledge and role as expert caregivers. “ They asked if she had a favorite toy … and we could play her music if we wanted” (1).

Parents noted that a non-sterile, child-friendly setting helped reduce the intimidating aspects of the monitored intensive care unit. Minor details in room design, such as colorful or personalized spaces, provided a sense of familiarity and contributed to emotional adjustment in an otherwise highly monitored environment. “And then I think the environment there also plays a big role, that it wasn't so sterile, but it was.. it was child-friendly” (2). Thoughtful actions such as providing space to sit, blankets, or being asked about preferences in care tasks contributed to a sense of inclusion and safety.

## 4 Discussion

To our knowledge, this qualitative exploratory study is the first to examine how vital sign monitoring influences parents’ perceived participation in the care of children with CHD. The findings are captured within the overarching theme striving for connection and control through participation and support in a monitored care environment, comprising three main categories and six subcategories. The results provide new insights into what it means to be a parent in an unfamiliar, technology-intensive care context. They illustrate how monitoring affects parents as individuals, how parents manage the physical and emotional distance that monitoring may create between themselves and their child, and how healthcare professionals play a crucial role in establishing conditions that support parental participation and reinforce parents' sense of responsibility in their parenting role. Parents in this study experienced monitoring as both hindering and facilitating their opportunities to participate in care and meet their children's needs. To remain actively involved, parents emphasized the importance of receiving prior information about situations that could trigger alarms, including false alarms or atypical readings. Delayed information, information that did not align with actual events, or sudden changes in circumstances often generated worry rather than reassurance. Parents described how their sense of participation in the monitoring process developed gradually over time, supported by guidance and information from healthcare professionals. This aligns with previous research indicating that clear communication and active involvement improve parents' understanding of the purpose and function of monitoring and can transform technology from a source of fear into a tool that promotes reassurance and participation (19, 31). In the present study, ongoing information and clear, consistent explanations enabled parents to feel included in decision-making, enhanced their sense of reassurance, and improved their ability to cope with the unfamiliar high-technology care environment, thereby reducing fear and uncertainty. Consistent with the findings of Engström et al. (32), parents described how engagement with monitoring supported their sense of control by allowing them to focus on their child's vital signs and anticipate both improvements and deterioration, which contributed to a sense of security. At the same time, parents expressed a strong need for unrestricted access to their child to prevent feelings of loss of control. The closely monitored hospital environment was both reassuring and challenging. While parents acknowledged that continuous monitoring was essential for their child's safety, the equipment also created physical barriers that contributed to feelings of separation. Alarms and monitoring devices served as constant reminders of the child's vulnerability, and reliance on healthcare professionals to lift or reposition the child led to feelings of frustration and helplessness. Previous studies have shown that parents of children cared for in pediatric intensive care units (PICUs) often experience a loss of their parental role (16, 19, 33). The physical and symbolic presence of monitoring technology can limit touch and intimacy, creating emotional distance between parent and child (16, 17, 34). The findings of the present study indicate that guidance and support from healthcare professionals strengthened parents' sense of parental competence and mitigated feelings of being overwhelmed. Active participation enabled parents to maintain closeness to their child and focus on providing comfort and emotional support. To cope with the situation, parents emphasized the need for support not only for their child but also for themselves and other family members involved in caregiving. Similar findings were reported by Poole et al. (16), where parents recognized the importance of attending to their own needs and actively sought emotional and practical support. An essential form of support identified in this study was parents' ability to leave the ward to rest and recover psychologically. From a participatory perspective, this suggests that when parents feel informed and maintain a sense of control over their child's care, they feel safe entrusting their child to healthcare professionals for short periods. Previous research has shown that the high-technology PICU environment may lead parents to withdraw as a coping strategy, creating physical, emotional, or spiritual distance as a means of self-protection when stress becomes overwhelming (15, 31). Overall, the findings illuminate parents' needs and strategies for participation in a monitored care environment. Information and support from healthcare professionals enabled parents to understand the monitoring process, feel involved in decision-making, and maintain a sense of parental responsibility. Both parental participation and support for siblings were largely dependent on healthcare professionals' actions and attitudes during monitoring. These findings underscore the importance of adopting a family-centered care approach, in which parents are recognized as essential partners in their child's care (22), even within highly technological care environments. This study contributes new knowledge on how vital sign monitoring in children undergoing surgery for CHD affects parents' experiences of participation and their need for involvement in care.

### 4.1 Strengths and limitations

A key strength of qualitative research is its ability to capture participants lived experiences in depth (27, 35). The length of the interviews may be considered a limitation, as they were relatively short. However, in qualitative research, the adequacy of data is not determined solely by interview duration, but by the relevance and richness of the information obtained. The concept of information power suggests that studies with a narrow aim and a specific sample may require fewer and shorter interviews to achieve sufficient depth (36). Given the focused aim of exploring parental participation in relation to monitoring the child's vital parameters, the interviews were considered to provide sufficiently rich and relevant data (37).

By focusing on parents' experiences, this study contributes insights into how participation is perceived and enacted, monitored perioperatively in a context. To enhance trustworthiness, purposive sampling was used to recruit participants with relevant and varied experiences. Although this approach may have limited the diversity of perspectives, it ensured the inclusion of participants with meaningful experiences aligned with the study aim. In line with the concept of information power, the relatively homogeneous sample supports that the sample size and data were sufficient to generate trustworthy findings (36, 38).

The sample in this study consisted primarily of mothers and mainly reflects maternal perspectives. The caregiving responsibilities and parental experiences can differ between mothers and fathers, particularly in relation to emotional and practical responsibility. However, one father was included, which can increase the transferability of the findings (39).

All interviews were conducted by the second author, with the help of a digital meeting platform (Zoom), which may have influenced the quality of interactions due to the lack of in-person meetings. However, this approach facilitated participation by allowing parents from different geographic areas to schedule interviews based on their availability. All authors were involved in the analysis and had different backgrounds and pre-understanding (registered nurses with varying specialist nursing qualifications) (36). The integration of researchers with different backgrounds and pre-understanding can be considered a strength, due to the various interpretations from diverse perspectives. Quotations were used in the result, which could also strengthen credibility (39). Sufficient information, such as a detailed description of the setting, study participants, and the analysis process can strengthen the transferability of the study (39). It should be noted that the setting, i.e., the national healthcare unit, may differ in design and function from an international perspective. However, it is the responsibility of the reader to determine whether the findings are transferable to other contexts. Nevertheless, some important limitations need to be highlighted. For instance, member checks were not possible due to logistical issues. According to Lloyd et al. (40), decisions regarding member checking should be based on methodological considerations, and the absence of this step does not inherently affect credibility. The data collection period extended over more than one year (March 2024-January 2025), which may be considered a limitation due to contextual or organizational factors that might have changed during this time. However, the consistency of the findings across the interviews suggests that the participants' experiences were fairly stable (35)..

### 2.2 Implications for practice

Consistent with the study's findings, healthcare professionals should prioritize clear communication about monitoring procedures, creating space for parental questions, and encouraging meaningful physical and emotional proximity between parents and their child. Having parents nearby requires awareness of their vulnerability in the high-technology care environments where their child's vital signs are monitored. The individual care plan for the child should explicitly include this awareness. Parents should be treated individually and provided with support that helps them feel responsible and confident in their parenting role, while being allowed to remain close to their child and, when necessary to feel comfortable stepping away.

### 2.3 Further research

Further research is warranted to examine how healthcare professionals can support and facilitate parental participation during vital sign monitoring in ways that are tailored to the individual needs of parents of children with CHD, ranging from being present to active involvement in care and decision-making.

## 5 Conclusion

This study contributes to an understanding of how a child's care during pre- and postoperative monitoring of vital signs affects parentś perceived participation. It emphasizes the need for care practices that preserve parental engagement and emotional contact in technologically advanced care settings. To facilitate meaningful participation in line with family-centered care, parents should be supported in looking beyond the technical aspects of care. Such support is essential to enable parents to assume as much responsibility as possible for their child's care, while ensuring that both the child's and the parents' need for closeness is met. Healthcare professionals play a key role in this process by actively involving parents and other family members, providing tailored information, and creating opportunities for individual questions. However, it is important to recognize that parents cannot and should not participate in their child's monitoring at the same level as healthcare professionals, which may present challenges. In this context, it is also essential to continuously evaluate and adjust the intensity of monitoring.

## Data Availability

The datasets presented in this article are not readily available because the qualitative data upon which this analysis was conducted is not publicly available due to ethical concerns regarding confidentiality of participants. Further, consent was not obtained from participants to share information from interview transcripts with third parties not involved in the research and the ethical approval for this study doesn’t permit the sharing of such information. Requests to access the datasets should be directed to carina.sjoberg@med.lu.se.
