# Supplementary material for: Parental participation during children’s vital sign monitoring in connection with surgery for congenital heart disease
Source: Front Pediatr. 2026 Jun 9;14:1809877. doi: 10.3389/fped.2026.1809877 (PMC13286857; doi:10.3389/fped.2026.1809877)
Supplement: Supplementary file 1 [file Datasheet1.pdf]

Interview Guide – Parents

Interview study for parents of children from newborn to 36 months who are being or have been monitored before and after surgery with medical-technical monitoring.

The concept of participation: Active involvement, being informed, having influence. This creates a sense of being needed and useful – participation.

Can you tell me about your experience with your child being monitored using medical technology before and after surgery?

What did you think?

What did you feel?

What did you do?

What needs did/do you have as a parent in connection with your child being monitored?

Was there anything you missed?

Was there anything that would have made it easier for you?

Was there anything that helped you feel involved?

Was there anything that hindered your participation/active involvement?

In what way did it help you or hinder you?

What is meaningful for you as a parent to feel needed?

What makes you as a parent feel that you are not needed?

Information: Where, when, how, level, timing of information? Relationship with healthcare staff: Attitude, demands from staff? Environment: Possibilities for distraction, waiting.

Sjöberg, C., Svedberg, P., Nygren, JM., Carlsson, IM. (2017). Participation in pediatric perioperative care: ` what it means for parents. Journal of Clinical Nursing, 2. https://doi.org/10.1111/jocn.13747
